# Supplementary material for: Predict models for prolonged ICU stay using APACHE II, APACHE III and SAPS II scores: A Japanese multicenter retrospective cohort study
Source: PLoS One. 2022 Jun 16;17(6):e0269737. doi: 10.1371/journal.pone.0269737 (PMC9202898; doi:10.1371/journal.pone.0269737)
Supplement: Attachment — Submitted filename: Response to Reviewers.docx [file pone.0269737.s001.docx]

April 4, 2022

Submission no: PONE-D-22-01670

Submission title: Predict models for prolonged ICU stay using APACHE II, APACHE III and SAPS II scores: A Japanese multicenter retrospective cohort study

Dear Academic Editor Yu Ru Kou, PhD

Thank you for your letter of March 30 regarding the above manuscript. We are pleased to know that our manuscript will be reconsidered after revision according to your reviewers’ comments. Our responses to the reviewers’ comments are noted below.

Reviewer #1:

Major

Q1. Table 1 looks so long and contained too many information. I recommend to divide the table 1 appropriately like backgrounds including number, male, age, BMI, kinds of admission, outcome as 1 table, and diagnosis and chronic disease as 1 table, interventions as 1 table, and independent 1 table for about APACHE2, 3, and SAPSII.

R1. As suggested, we divided the Table 1 to Table 1–4.

Q2．The author succussed to approve models for predicting prolonged stays in Japanese ICU with appropriate statistical analysis, however there are too many long tables to understand not easy. I recommend two figures to show AUC for predicting prolonged ICU stay and hospital death with each model, if the author will accept and consider looking better. It’s up to the author.

R2. As suggested, we added Fig 3, 4 to show ROC curves for predicting prolonged ICU stay and hospital death with each model.

Minor

Q1．Please add more detail explanation about JIPAD like as the number of participated facilities, on line-based input system, and past achievements, in page 4, lines 25 to Page4, line 2.

R1. As suggested, we added more detail explanation about JIPAD in the materials and methods section (page 4, line 15–21). Detail explanation about JIPAD is below.

JIPAD was established by the Japanese Society of Intensive Care Medicine in 2014. 94 ICUs participate in JIPAD as of December 31, 2021. Each participating ICU submits data of their ICU patients, such as characteristics, severity scores at ICU admission, therapies in the ICU and outcomes, using on-line data system. Submitted data are routinely monitored and corrected by members of the JIPAD working group to improve the credibility of data. A recent study developed a mortality prediction model for adult patients admitted to ICU in Japan using JIPAD [13]. Further information about JIPAD can be found elsewhere [12].

12. Irie H, Okamoto H, Uchino S, Endo H, Uchida M, Kawasaki T, et al.; JIPAD Working Group in the Japanese Society of Intensive Care Medicine. The Japanese Intensive care PAtient Database (JIPAD): A national intensive care unit registry in Japan. J Crit Care. 2020; 55: 86-94. doi: 10.1016/j.jcrc.2019.09.004.

13. Endo H, Uchino S, Hashimoto S, Aoki Y, Hashiba E, Hatakeyama J, Hayakawa K, Ichihara N, Irie H, Kawasaki T, Kumasawa J, Kurosawa H, Nakamura T, Ohbe H, Okamoto H, Shigemitsu H, Tagami T, Takaki S, Takimoto K, Uchida M, Miyata H. Development and validation of the predictive risk of death model for adult patients admitted to intensive care units in Japan: an approach to improve the accuracy of healthcare quality measures. J Intensive Care. 2021; 9(1): 18. doi: 10.1186/s40560-021-00533-z.

Q2. Please add full spell of MV as “mechanical ventilation” in the Page5, line 6.

R2. We are sorry for this mistake. We added it.

Q3. Please re-check the expression of page in each reference of NO. 6 and 18.

R3. We are sorry for this mistake. We corrected it.

Reviewer #2:

Major comments

Q1. First, author should mention about the definition of prolonged ICU stay. Why did you chose 14 days ? Author must show the definition and its reason, not only referring just citations.

R1. As suggested, we added the reason why we chose this definition in the materials and methods section (page 4, line 26 to page 5, line 3). The reason is below.

It is because special fees for critical care are limited to a period of 14 days in the Japanese health insurance system, even if the status of the patient remains serious for more than 14 days. Additionally, previous studies about prolonged ICU stay that conducted in other countries also used this definition [2,14,15].

2. Arabi Y, Venkatesh S, Haddad S, Al Shimemeri A, Al Malik S. A prospective study of prolonged stay in the intensive care unit: predictors and impact on resource utilization. Int J Qual Health Care. 2002; 14(5): 403-10. doi: 10.1093/intqhc/14.5.403.

14. Laupland KB, Kirkpatrick AW, Kortbeek JB, Zuege DJ. Long-term mortality outcome associated with prolonged admission to the ICU. Chest. 2006; 129(4): 954-9. doi: 10.1378/chest.129.4.954.

15. Zampieri FG, Ladeira JP, Park M, Haib D, Pastore CL, Santoro CM, et al. Admission factors associated with prolonged (>14 days) intensive care unit stay. J Crit Care. 2014; 29(1): 60-5. doi: 10.1016/j.jcrc.2013.09.030.

Q2. It is too difficult for clinician to understand usefuleness for this predictive models. After all, which combination of factors is the best for predicting?

R2. Thank you for your variable comment. As you pointed out, there are a lot of variables in our models. Thus, we performed backward deletion to reduce variables in a stepwise manner. The results are shown in below table (Model 1–3). However, only “Male” was removed from each model, so our models still contained seven variables. Generally, predict models are not simple. Indeed, although previous studies also developed predict models for prolonged ICU stay, their models contain a lot of variables as well as the present study (2, 16). It is because these studies didn't intend to develop simple predict models but intended to develop accurate predict models as well as our study. Indeed, the AUC values for all our predict models were more than 0.8, which means that the discrimination abilities of these models were good. So, we did not change the table 5. The present models look complicated due to many categories, particularly in diagnosis, but there are only eight variables in these models. It is not so complicated.

| Model 1 |  |  |  |
| --- | --- | --- | --- |
|  | aOR | 95% CI | P value |
| (Intercept) | 0.007 | 0.006, 0.010 | <0.001* |
| APACHE II |  |  |  |
| –10 | reference |  |  |
| 11–20 | 3.650 | 2.910, 4.590 | <0.001* |
| 21–30 | 12.60 | 9.940, 15.90 | <0.001* |
| 31–40 | 17.90 | 13.80, 23.00 | <0.001* |
| 41+ | 9.050 | 6.500, 12.60 | <0.001* |
| BMI (kg/m^2^) |  |  |  |
| 18.5 ≤, < 25 | reference |  |  |
| < 18.5 | 0.953 | 0.848, 1.070 | 0.419 |
| 25 ≤, < 35 | 1.140 | 1.030, 1.270 | 0.013* |
| 35 ≤ | 1.330 | 0.936, 1.890 | 0.111 |
| RRT/MET | 1.240 | 1.060, 1.470 | <0.001* |
| Postresuscitation | 1.330 | 1.090, 1.610 | <0.001* |
| Readmisson | 1.290 | 1.100, 1.500 | <0.001* |
| LOS before ICU admission (days) |  |  |  |
| 0 | reference |  |  |
| 1–7 | 0.757 | 0.674, 0.850 | <0.001* |
| 8–14 | 0.970 | 0.821, 1.150 | 0.724 |
| 15+ | 1.280 | 1.120, 1.470 | <0.001* |
| Diagnosis |  |  |  |
| -Post-operative |  |  |  |
| Cardiovascular disease | reference |  |  |
| Respiratory disease | 0.209 | 0.146, 0.301 | <0.001* |
| Digestive disease | 0.550 | 0.466, 0.650 | <0.001* |
| Neurological disease | 0.675 | 0.556, 0.820 | <0.001* |
| Trauma | 1.260 | 0.879, 1.800 | 0.208 |
| Metabolic/hematological disease | 0.425 | 0.158, 1.140 | 0.091 |
| Urinary disease | 0.145 | 0.071, 0.293 | <0.001* |
| Muscle/bone/skin disease | 0.282 | 0.185, 0.430 | <0.001* |
| Obstetrics/gynecological disease | 0.177 | 0.073, 0.429 | <0.001* |
| -Non-operative |  |  |  |
| Cardiovascular disease | 0.894 | 0.755, 1.060 | 0.200 |
| Respiratory disease | 2.080 | 1.790, 2.410 | <0.001* |
| Digestive disease | 1.170 | 0.950, 1.440 | 0.140 |
| Neurological disease | 1.280 | 1.040, 1.570 | 0.022* |
| Sepsis | 1.020 | 0.807, 1.280 | 0.882 |
| Trauma | 1.490 | 1.080, 2.050 | 0.014* |
| Metabolic disease | 0.333 | 0.214, 0.518 | <0.001* |
| Hematological disease | 1.133 | 0.698, 1.820 | 0.622 |
| Urinary disease | 0.779 | 0.456, 1.330 | 0.359 |
| Muscle/bone/skin disease | 2.030 | 1.380, 2.980 | <0.001* |
| Others | 1.260 | 0.790, 2.010 | 0.331 |

| Model 2 |  |  |  |
| --- | --- | --- | --- |
|  | aOR | 95% CI | P value |
| (Intercept) | 0.005 | 0.003, 0.006 | <0.001* |
| APACHE III score |  |  |  |
| –40 | reference |  |  |
| 41–80 | 6.270 | 4.820, 8.160 | <0.001* |
| 81–120 | 21.20 | 16.20, 27.80 | <0.001* |
| 121–160 | 28.50 | 21.30, 38.20 | <0.001* |
| 161+ | 12.50 | 8.530, 18.30 | <0.001* |
| BMI (kg/m^2^) |  |  |  |
| 18.5≤, < 25 | reference |  |  |
| <18.5 | 0.952 | 0.847, 1.070 | 0.410 |
| 25 ≤, < 35 | 1.160 | 1.040, 1.290 | 0.006* |
| 35 ≤ | 1.370 | 0.965, 1.950 | 0.078 |
| RRT/MET | 1.250 | 1.060, 1.480 | 0.007* |
| Postresuscitation | 1.250 | 1.020, 1.520 | 0.030* |
| Readmisson | 1.240 | 1.060, 1.440 | 0.007* |
| LOS before ICU admission (days) |  |  |  |
| 0 | reference |  |  |
| 1–7 | 0.774 | 0.689, 0.869 | <0.001* |
| 8–14 | 0.955 | 0.808, 1.130 | 0.593 |
| 15+ | 1.290 | 1.130, 1.480 | <0.001* |
| Diagnosis |  |  |  |
| -Post-operative |  |  |  |
| Cardiovascular disease | reference |  |  |
| Respiratory disease | 0.231 | 0.161, 0.332 | <0.001* |
| Digestive disease | 0.513 | 0.434, 0.607 | <0.001* |
| Neurological disease | 0.846 | 0.695, 1.030 | 0.095 |
| Trauma | 1.470 | 1.020, 2.100 | 0.004* |
| Metabolic/hematological disease | 0.511 | 0.189, 1.380 | 0.185 |
| Urinary disease | 0.156 | 0.078, 0.316 | <0.001* |
| Muscle/bone/skin disease | 0.285 | 0.187, 0.435 | <0.001* |
| Obstetrics/gynecological disease | 0.193 | 0.080, 0.468 | <0.001* |
| -Non-operative |  |  |  |
| Cardiovascular disease | 0.969 | 0.817, 1.150 | 0.717 |
| Respiratory disease | 2.270 | 1.950, 2.630 | <0.001* |
| Digestive disease | 1.200 | 0.972, 1.480 | 0.091 |
| Neurological disease | 1.430 | 1.160, 1.760 | <0.001* |
| Sepsis | 1.050 | 0.835, 1.330 | 0.663 |
| Trauma | 1.710 | 1.250, 2.360 | <0.001* |
| Metabolic disease | 0.360 | 0.232, 0.560 | <0.001* |
| Hematological disease | 1.210 | 0.749, 1.960 | 0.434 |
| Urinary disease | 0.817 | 0.478, 1.400 | 0.459 |
| Muscle/bone/skin disease | 2.080 | 1.410, 3.060 | <0.001* |
| Others | 1.450 | 0.906, 2.310 | 0.122 |

| Model 3 |  |  |  |
| --- | --- | --- | --- |
|  | aOR | 95% CI | P value |
| (Intercept) | 0.004 | 0.003, 0.005 | <0.001* |
| SAPS II score |  |  |  |
| –25 | reference |  |  |
| 26–50 | 7.590 | 6.200, 9.300 | <0.001* |
| 51–75 | 22.60 | 18.30, 28.00 | <0.001* |
| 76–100 | 21.30 | 16.60, 27.30 | <0.001* |
| 101+ | 6.830 | 4.090, 11.40 | <0.001* |
| BMI (kg/m^2^) |  |  |  |
| 18.5 ≤, < 25 | reference |  |  |
| < 18.5 | 0.963 | 0.856, 1.080 | 0.523 |
| 25 ≤, < 35 | 1.150 | 1,030, 1.270 | 0.001* |
| 35 ≤ | 1.370 | 0.965, 1.960 | 0.078 |
| RRT/MET | 1.220 | 1.030, 1.430 | 0.019* |
| Postresuscitation | 1.360 | 1.120, 1.660 | 0.002* |
| Readmisson | 1.200 | 1.030, 1.400 | 0.020* |
| LOS before ICU admission (days) |  |  |  |
| 0 | reference |  |  |
| 1–7 | 0.905 | 0.807, 1.010 | 0.086 |
| 8–14 | 1.120 | 0.945, 1.320 | 0.194 |
| 15+ | 1.520 | 1.320, 1.740 | <0.001* |
| Diagnosis |  |  |  |
| -Post-operative |  |  |  |
| Cardiovascular disease | reference |  |  |
| Respiratory disease | 0.298 | 0.207, 0.429 | <0.001* |
| Digestive disease | 0.575 | 0.486, 0.680 | <0.001* |
| Neurological disease | 0.862 | 0.707, 1.050 | 0.141 |
| Trauma | 1.270 | 0.885, 1.810 | 0.197 |
| Metabolic/hematological disease | 0.591 | 0.218, 1.600 | 0.300 |
| Urinary disease | 0.223 | 0.110, 0.450 | <0.001* |
| Muscle/bone/skin disease | 0.351 | 0.230, 0.537 | <0.001* |
| Obstetrics/gynecological disease | 0.267 | 0.110, 0.647 | <0.001* |
| -Non-operative |  |  |  |
| Cardiovascular disease | 0.915 | 0.774, 1.080 | 0.301 |
| Respiratory disease | 2.330 | 2.010, 2.700 | <0.001* |
| Digestive disease | 1.300 | 1.050, 1.590 | 0.014* |
| Neurological disease | 1.340 | 1.090, 1.650 | 0.006* |
| Sepsis | 1.150 | 0.913, 1.450 | 0.236 |
| Trauma | 1.620 | 1.180, 2.230 | 0.003* |
| Metabolic disease | 0.384 | 0.247, 0.597 | <0.001* |
| Hematological disease | 1.300 | 0.802, 2.100 | 0.289 |
| Urinary disease | 0.946 | 0.554, 1.620 | 0.840 |
| Muscle/bone/skin disease | 2.400 | 1.630, 3.540 | <0.001* |
| Others | 1.540 | 0.966, 2.450 | 0.069 |

2. Arabi Y, Venkatesh S, Haddad S, Al Shimemeri A, Al Malik S. A prospective study of prolonged stay in the intensive care unit: predictors and impact on resource utilization. Int J Qual Health Care. 2002; 14(5): 403-10. doi: 10.1093/intqhc/14.5.403.

16. Kramer AA, Zimmerman JE. A predictive model for the early identification of patients at risk for a prolonged intensive care unit length of stay. BMC Med Inform Decis Mak. 2010; 10: 27. doi: 10.1186/1472-6947-10-27.

Q3. Author should use more figures for understanding. I think that ROC analysis is suitable for this study. Please show visually ROC analysis by figure.

R3. As suggested (reviewer 1 also suggested), we added Fig 3, 4 to show ROC curves for predicting prolonged ICU stay and hospital death with each model.

We thank the reviewers for their comments and hope that this paper is now suitable for publication in PLOS ONE.

Sincerely yours,

Daiki Takekawa, M.D, PhD.
